# Diapocynin neuroprotective effects in 3-nitropropionic acid Huntington’s disease model in rats: emphasis on Sirt1/Nrf2 signaling pathway

**DOI:** 10.1007/s10787-022-01004-z

**Published:** 2022-05-31

**Authors:** Weam W. Ibrahim, Nora O. Abdel Rasheed

**Affiliations:** Present Address: Department of Pharmacology and Toxicology, Faculty of Pharmacy, Cairo University, Kasr El Aini St., Cairo, 11562 Egypt

**Keywords:** NADPH oxidase, Diapocynin, 3-nitropropionic acid, Nrf2, Huntington's disease, Sirt1

## Abstract

**Background and Aim:**

Huntington's disease (HD) is a rare inherited disease portrayed with marked cognitive and motor decline owing to extensive neurodegeneration. NADPH oxidase is considered as an important contributor to the oxidative injury in several neurodegenerative disorders including HD. Thus, the present study explored the possible neuroprotective effects of diapocynin, a specific NADPH oxidase inhibitor, against 3-nitropropionic acid (3-NP) model of HD in rats.

**Methods:**

Animals received diapocynin (10 mg/kg/day, p.o), 30 min before 3-NP (10 mg/kg/day, i.p) over a period of 14 days.

**Results:**

Diapocynin administration attenuated 3-NP-induced oxidative stress with significant increase in reduced glutathione, glutathione-S-transferase, nuclear factor erythroid 2-related factor 2, and brain-derived neurotrophic factor striatal contents contrary to NADPH oxidase (NOX2; gp91phox subunit) diminished expression. Moreover, diapocynin mitigated 3-NP-associated neuroinflammation and glial activation with prominent downregulation of nuclear factor-Кβ p65 and marked decrement of inducible nitric oxide synthase content in addition to decreased immunoreactivity of ionized calcium binding adaptor molecule 1 and glial fibrillary acidic protein; markers of microglial and astroglial activation, respectively. Treatment with diapocynin hindered 3-NP-induced apoptosis with prominent decrease in tumor suppressor protein and Bcl-2-associated X protein contents whereas the anti-apoptotic marker; B-cell lymphoma-2 content was noticeably increased. Diapocynin neuroprotective effects could be attributed to silent information regulator 1 upregulation which curbed 3-NP-associated hazards resulting in improved motor functions witnessed during open field, rotarod, and grip strength tests as well as attenuated 3-NP-associated histopathological derangements.

**Conclusion:**

The present findings indicated that diapocynin could serve as an auspicious nominee for HD management.

**Graphical abstract:**

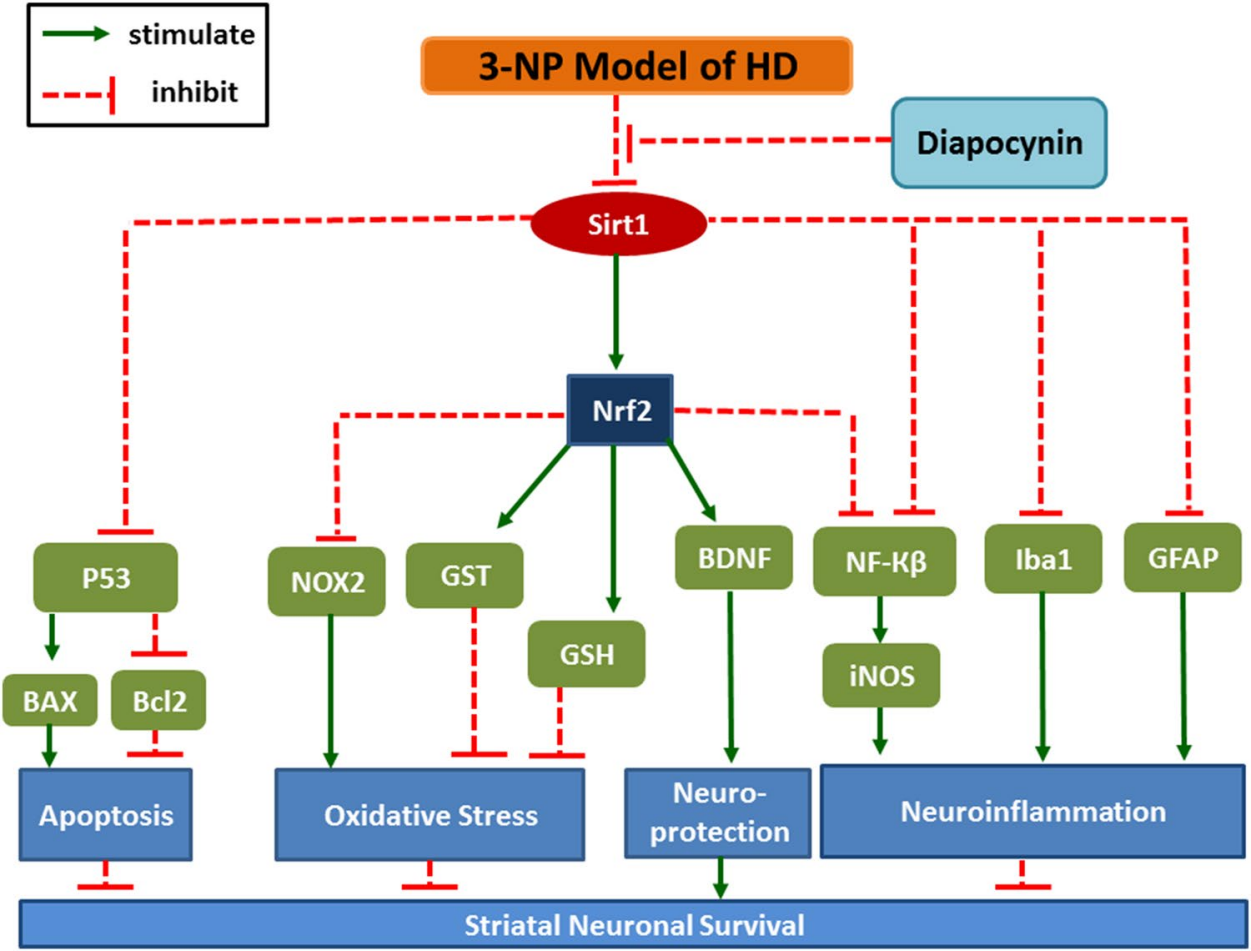

## Introduction

Huntington’s disease (HD) is a devastating autosomal inherited neurodegenerative disease. It is distinguished by a triad of motor dysfunction, psychiatric disruption, and cognitive dysfunction. Neuronal loss especially in the striatum of HD patients is the major feature of the disease as the striatum is involved in motor control as well as learning functions (Wiprich and Bonan 2021). HD prevalence is about 5 cases in 100,000 people (Illarioshkin et al. 2018). The available treatments for HD provide only symptomatic relief with numerous undesirable side effects; thus, it is essential to investigate safer and more effective therapies that can halt the disease progression (Kim et al. 2017).

Administration of 3-nitropropionic acid (3-NP), a selective striatal neurotoxin, is reported to mimic neuropathological changes of HD as it inhibits succinate dehydrogenase enzyme leading to oxidative stress (Damiano et al. 2010; Zuccato et al. 2010). Moreover, 3-NP promoted microglial activation evidenced by the prominent increase of ionized calcium binding adaptor molecule 1 (Iba1)-immunoreactive cells, along with augmented release of inflammatory cytokines that contribute to neurological dysfunction and striatal neuronal death (Kim et al. 2017). Nicotinamide dinucleotide phosphate (NADPH) oxidase (NOX), a crucial source of reactive oxygen species (ROS), was witnessed at high levels in the striatum of HD patients especially NOX2 isoform. Moreover, NOX activity as well as ROS-associated neuronal death were prominently decreased upon treatment of HD patients with NOX inhibitors (Valencia et al. 2013). The nuclear factor erythroid 2-related factor 2 (Nrf2) signaling pathway is implicated in the downregulation of nuclear factor-Кβ (NF-Кβ) and its downstream inflammatory cytokines which contribute to many neurodegenerative disorders such as HD (Memet 2006). Administration of 3-NP is associated with Nrf2 downregulation leading to augmented oxidative stress, neuro-inflammation, and apoptosis (Kim et al. 2017). Silent information regulator 1 (Sirt1) is involved in the induction of Nrf2 transcriptional activity as well as the expression of its downstream genes as those related to reduced glutathione (GSH), the well-known redox scavenger (Ren et al. 2019). Additionally, neuroprotective effect of Sirt1 was correlated with induction of brain-derived neurotrophic factor (BDNF) expression (Tian et al. 2020). BDNF is a principle neuroprotective factor that is reported to induce neurogenesis and combat apoptosis, in addition to its imperative role in synaptic plasticity (Xia et al. 2010). Moreover, Sirt1 is known to impede tumor suppressor protein (P53) activity along with the inhibition of its downstream genes expression such as Bcl-2-associated X protein (BAX), thus hindering apoptosis (Rahman and Islam, 2011; Ren et al. 2019). 3-NP model of HD is associated with prominent decrease in Sirt1 and BDNF expression which contributes to neuronal damage witnessed in this model (Sayed et al. 2020). It is also reported that 3-NP is implicated in P53 upregulation with increased expression of pro-apoptotic proteins as BAX triggering apoptotic cell death (Gopinath et al. 2011; Gonchar et al. 2021).

Diapocynin is an oxidative derivative of the naturally occurring agent apocynin, the most commonly used investigational NOX inhibitor. Compared to apocynin, diapocynin is found to possess higher lipophilicity and greater potency to inhibit NOX activity (Luchtefeld et al. 2008; Kanegae et al. 2010). It is supposed to inhibit NOX by hindering the migration of the enzyme cytosolic subunits to the membrane as well as their binding with membrane components, and thus preventing the enzyme activation and the subsequent generation of superoxide anion and the other hazardous ROS (Stolk et al. 1994). Diapocynin has shown anti-oxidant, anti-inflammatory, and neuroprotective properties against 1-methyl-4-phenyl-1,2,3,6-tetrahydropyridine (MPTP) mouse model of Parkinson's disease and d-galactose/ovariectomy rat model of Alzheimer's disease primarily via NOX inhibition (Ghosh et al. 2012; Ibrahim et al. 2020). Apocynin has been found to exhibit powerful anti-oxidant and anti-inflammatory effects in various *in vitro* and *in vivo* models of neurodegenerative diseases including rotenone-induced Parkinson's disease (Gao et al. 2003), transgenic mouse model of Alzheimer's disease (Lull et al. 2011), focal or global cerebral ischemia (Gu et al. 2005; Jackman et al. 2009), familial amyotrophic lateral sclerosis model (Harraz et al. 2008), pilocarpine model of epilepsy (Pestana et al. 2010), and ketamine-induced psychosis (Behrens et al. 2007). Moreover, apocynin was found to attenuate motor alterations assessed as circling behavior and to mitigate the striatal neuronal damage in HD rat model induced by intra-striatal injection of quinolinic acid (Maldonado et al. 2010). Therefore, the present study was performed to explore the possible neuroprotective effects of diapocynin in 3-NP model of HD focusing on the involvement of Sirt1-mediated signaling pathways.

## Materials and methods

### Animals

Adult male Wistar rats weighing 180 ± 20 g were purchased from the animal facility of Faculty of Pharmacy, Cairo University (Cairo, Egypt). They were acclimated to the laboratory circumstances for 2 weeks before the study beginning. Rats were kept under controlled environmental conditions of constant temperature (23 ± 2 °C), humidity (60 ± 10%), and light/dark (12/12 h) cycle. Food and water were freely permitted during the experiment. This study was carried out in line with the principles of *The Guide for Care and Use of Laboratory Animals* published by the US National Institutes of Health (NIH Publication No. 85–23, revised 2011) and was approved by the Ethical Committee for Animal Experimentation of the Faculty of Pharmacy, Cairo University, with permit number PT 3162. All efforts were exerted to reduce the animal suffering through the experimental duration.

### Drugs and chemicals

Diapocynin and 3-NP (Sigma-Aldrich Chemical Co., MO, USA) were dissolved in 1% DMSO and saline, respectively (Kim et al. 2017; Ibrahim et al. 2020). The doses were freshly prepared every day.

### Experimental design

Animals were randomly categorized into four groups (15 rats each) as follows:

Group 1 received normal saline intraperitoneally as well as 1% DMSO orally and served as control group. Rats in group 2 received diapocynin orally at a dose of 10 mg/kg/day. The dose of diapocynin was chosen based on prior studies which have utilized diapocynin or its parent drug “apocynin” at a comparable dose range in D-galactose/ovariectomy (Ibrahim et al. 2020) as well as transgenic mouse models of Alzheimer's disease (Lull et al. 2011), and in chronic cerebral hypoperfusion model of vascular dementia (Choi et al. 2014). Group 3 was injected with 3-NP (10 mg/kg/day; i.p.) (Ross et al. 2014). In group 4, rats were administered diapocynin 30 min before 3-NP injection (Joseph et al. 2017). Drugs were given for 14 days and rats were subjected to the behavioral tests on the 15th day. Animals were then euthanized, and brains were dissected with striata being separated. Brain samples were processed for the histopathological and immunohistochemical examinations in addition to the estimation of biochemical parameters in the striatal region.

### Behavioral assessment

Open field, rotarod, and grip strength tests were used to measure the animals' motor functions 24 h after the last day of injection and they were conducted in the same order with a 2-h resting interval between the tests. ANY-Maze video tracking software (Stoelting Co, USA) was used to record the rats' movement in the open field apparatus.

#### Open field test

The animal’s impulsive locomotor functions were detected using a square wooden box (80 × 80 × 40 cm) apparatus with red walls and black polished floor having 16 equal squares distributed by means of white lines. Rats' behavior was recorded using an overhead camera located in the sound isolated room where the test was carried out. Each animal was kept in the center of the box to spontaneously explore the field for 3 min. The subsequent parameters were measured during the assigned time; distance traveled, mean speed, immobility time in addition to distance traveled and time active in the central squares and the corners as well as the number of rearing (Avila et al. 2010; Abdel Rasheed and Ibrahim 2022).

#### Rotarod test

This test is performed to assess the animals' motor co-ordination and balance. Each animal was subjected to 5-trial days before the beginning of the experiment. Animals showing the capability to remain on the rod (120 cm long and 3 cm in diameter with 20 rpm rotation speed) for 5 min were enrolled in the current investigation. The fall off latency was detected on the test day following the open field analysis (Sayed et al. 2020).

#### Grip strength test

The latency of gripping a horizontal wire was considered as an indirect measure of grip strength. Each rat was permitted to hang using its forepaws on a steel wire; 2 mm in diameter, 35 cm in length, which was strained horizontally at a height of 50 cm over a cushion support. The time taken by each animal while it is still holding the wire was recorded (Elbaz et al. 2018).

### Biochemical measurements

#### Enzyme-linked immunosorbent assay

Striata were dissected and homogenized to prepare 10% homogenates in saline. Afterwards, centrifugation was carried out and the protein content was assessed in the supernatants according to Lowry et al. (1951). The supernatants were also used to assess the following parameters, each according to the instructions of its corresponding ELISA kit; GSH (BlueGene Biotech CO., LTD, ShangHai, China), glutathione-S-transferase (GST), Nrf2, BDNF, and inducible nitric oxide synthase (iNOS) (My BioSource Inc., San Diego, CA, USA). Moreover, ELISA kits were also used to measure striatal contents of P53 (AFG Bioscience LLC, Northbrook, IL, USA), B-cell lymphoma-2 (Bcl2) (Cusabio, Wuhan, China), and BAX (Wuhan Fine Biotech Co., Wuhan, China).

#### Western blot analysis of Sirt1 and NF-Кβ p65

Following protein extraction from striatal tissues, equal protein volumes were segregated according to their molecular weight by sodium dodecyl sulfate-polyacrylamide gel electrophoresis and then transferred to a nitrocellulose membrane by the aid of a semi-dry transfer apparatus (Bio-Rad, Hercules, CA, USA). Membranes were placed in 5% blocking solution composed of non-fat milk to prevent non-specific binding. Afterwards, they were incubated overnight at 4 °C on a roller shaker with the subsequent primary antibodies: anti-β-actin (1:1000; Cat. No. PA5-16914), anti-Sirt1 (1:1000; Cat. No. PA5-20964), and anti-ρS536-NF-Кβ p65 (1:1000; Cat. No. PA5-17782) (ThermoFisher Scientific, MA, USA). Afterwards, membranes were washed and incubated with horseradish peroxidase-conjugated secondary antibody (Dianova, Hamburg, Germany). Finally, chemiluminescence was identified via the Amersham detection kit as designated by the manufacturer and subjected to X-ray film. A scanning laser densitometer (Biomed Instrument Inc., CA, USA) was used to quantify the target proteins and the results were normalized against β-actin protein expression.

#### Quantitative real-time PCR analysis of NOX2 subunit (gp91phox)

Using RNeasy Mini kit (Qiagen, Venlo, Netherlands), total RNA was separated from striatal tissues and its purity was assessed spectrophotometrically by 260/280 nm. The reverse transcription of the extracted RNA into cDNA was developed by the aid of Reverse Transcription System (Promega, Leiden, Netherlands) according to the kit's instructions. The gene expression of gp91phox was measured by the means of quantitative real-time PCR using SYBR Green Master Mix (Applied Biosystems, CA, USA). Table 1 displays the sequences of primers used. The relative expression of target genes was attained by the help of the 2^−ΔΔCT^ formula with β-actin being a reference gene (Livak and Schmittgen 2001).

**Table 1:** Sequences of the primers used for quantitative real-time PCR analysis

gp91phox	Forward; 5′-CGGAATCCTCTCCTTCCT-3′ Reverse; 5′-GCATTCACACACCACTCCAC-3′
β-actin	Forward; 5′-GGAGATTACTGCCCTGGCTCCTA-3′, Reverse; 5′-GACTCATCGTACTCCTGCTTGCTG-3′

### Histopathological examination

The whole brains were dissected and fixed in 10% neutral buffered formalin for 72 h. Samples were processed in serial grades of ethanol, cleared in xylene, and then they were infiltrated and embedded into Paraplast tissue embedding media. 5μm thick serial sagittal brain sections were cut by a rotatory microtome for demonstration of striatal regions in different samples and mounted on glass slides. Tissue sections were stained by Hematoxylin and Eosin (H&E) as a general staining method for microscopic examination using light microscope (Culling 2013). Nissl staining was carried out to identify surviving neurons with Nissl's granules as it indicates healthy tissues. Sections were deparaffinized and rehydrated with xylene and graduated alcohol series. Afterwards, they were stained in Nissl stain for 5 min and air dried at room temperature for 1 h. After being dipped in alcohol for a while, the sections were cleared with xylene, cover slipped and examined microscopically. Average number of intact neurons was estimated from six randomly selected areas for each section using the Leica Qwin software (Leica Microsystems GmBH, Wetzlar, Germany). Neurons with a visible nucleus as well as an apparent whole outline were designated as normal ones (Culling 2013).

### Immunohistochemical examination of Iba1 and GFAP

According to the manufacturer's protocols and instructions, immunohistochemical staining for Iba1 and glial fibrillary acidic protein (GFAP) was performed. Antigen retrieved brain tissue sections were blocked by 3% hydrogen peroxide for 15 min then they were incubated with the primary antibodies; anti-GFAP (Cat. No. 13-0300; Thermo Scientific Co., MA, USA; 1:100) and anti-Iba1 antibodies (Cat. No. ab108539; Abcam, MA, USA; 1:100) overnight at 4°C. Afterwards, they were washed with phosphate-buffered saline then incubated for 20 min with biotinylated link antibody and peroxidase-labeled streptavidin (Dako, Carpinteria, CA, USA). The reaction was visualized with 3,3′-diaminobenzidine tetrahydrochloride (DAB Substrate Kit, Vector Laboratories Inc., Burlingame, CA, USA). Sections were counterstained with hematoxylin, dehydrated, and cleared in xylene then examined via light microscope.

For immunohistochemical quantitative analysis, six random striatal non overlapping fields were scanned and analyzed for determining the positive mean area percentage of GFAP immunoexpression and the mean number of Iba1-positive microglial cells in each immunostained tissue section. All morphological examinations, photographs as well as quantitative analysis were recorded using Leica Application system modules for histological analysis (Leica Microsystems GmbH, Wetzlar, Germany).

### Statistical analysis

The study results were illustrated as mean ± S.E.M. The data were analyzed by the means of one-way analysis of variance test (one-way ANOVA) followed by Tukey’s multiple comparison test for all parameters excluding the rearing frequency which was evaluated using Kruskal–Wallis test followed by Dunn’s multiple comparison test and presented as median (max–min). Statistical analysis was implemented using GraphPad Prism software (version 6) with the level of significance being fixed at *p* < 0.05.

## Results

### Diapocynin alleviated 3-NP-induced behavioral and motor dysfunction in open field, rotarod, and grip strength tests

**Fig. 1:**
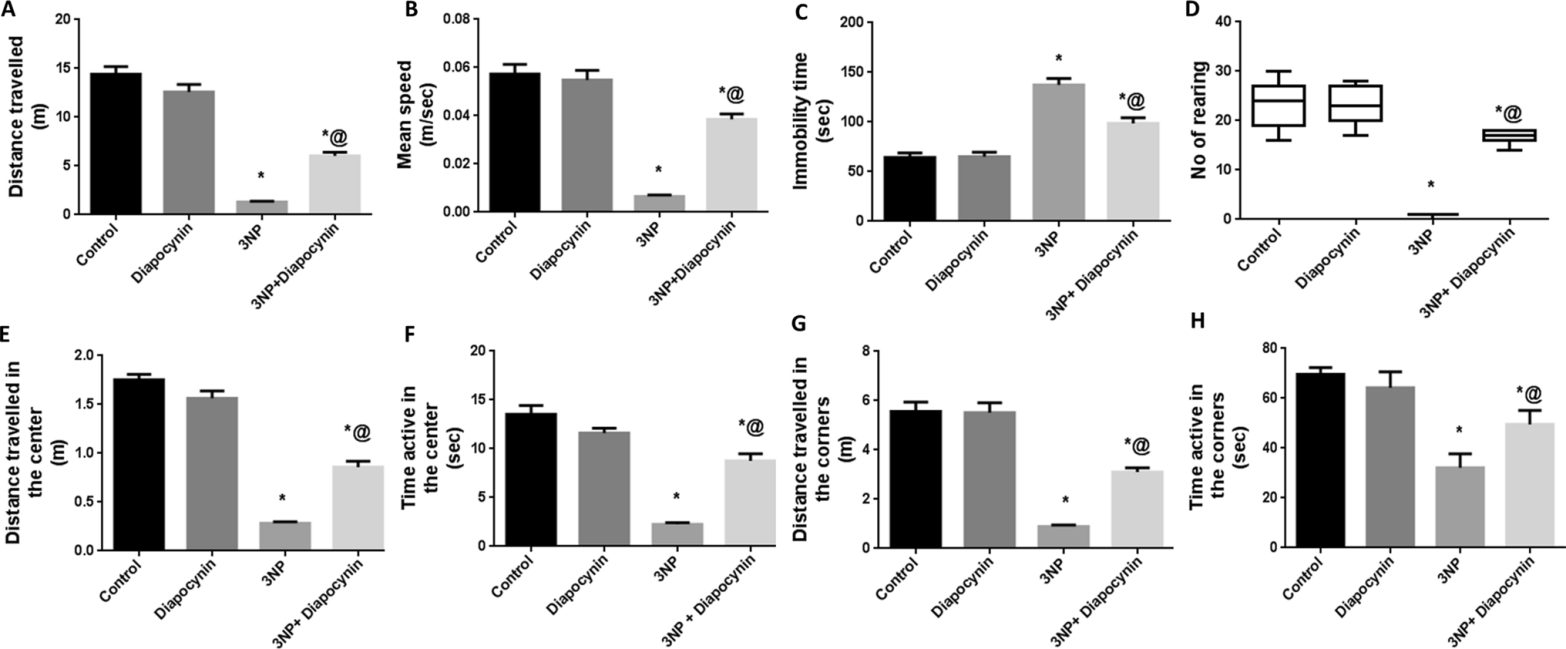
Effect of diapocynin on 3-NP-induced locomotor dysfunction witnessed in open field test. **A** Distance traveled, **B** Mean speed, **C** Immobility time, **D** Number of rearing, **E** Distance traveled in the center, **F** Time active in the center, **G** Distance traveled in the corners, and **H** Time active in the corners. Data were expressed as mean ± S.E.M. (*n* = 15). Statistical analysis was done using one-way ANOVA followed by Tukey’s post hoc test except for the number of rearing where data were presented as median (max–min) and analyzed using Kruskal–Wallis test followed by Dunn’s multiple comparison test. ^*^p < 0.05 vs. control, ^@^*p* < 0.05 vs. 3NP group. *3-NP* 3-nitropropionic acid

**Fig. 2:**
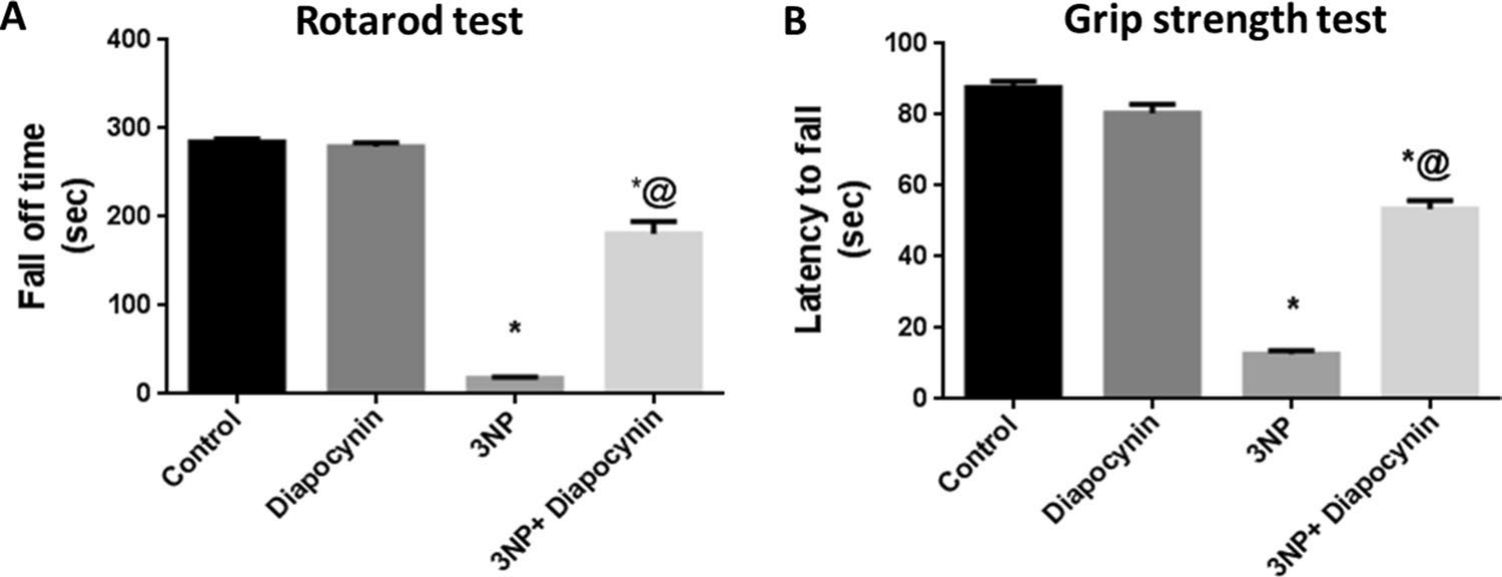
Effect of diapocynin on 3-NP-induced motor incoordination and muscle strength deficit regarding **A** Fall off time in rotarod test and **B** Latency to fall in grip strength test. Data were expressed as mean ± S.E.M. (*n* = 15). Statistical analysis was done using one-way ANOVA followed by Tukey’s post hoc test. ^*^p < 0.05 vs. control, ^@^*p* < 0.05 vs. 3NP group. *3-NP* 3-nitropropionic acid

Administration of 3-NP instigated profound locomotor dysfunction along with diminished muscle strength. Rats which received 3-NP have shown significant decrease of the total distance traveled (91%), mean speed (89%), and rearing frequency (96%) in open field test, as compared to the control group rats, F(3, 56) = 103.8 for total distance traveled and 57.35 for mean speed (*p* < 0.0001). Additionally, these animals exhibited diminished central and peripheral locomotor activities as evidenced by meaningfully reduced distance as well as time active in the center (84 and 83%, respectively) and the periphery (84 and 54%, respectively), contrary to increased immobility time (2.1-fold), as compared to normal control group; *F*(3, 56) = 150, 59.93, 58.97, 79.01, and 39.79, respectively (*p* < 0.0001). Meanwhile, 3-NP-injected rats displayed obvious reduction in the fall off latency (94%) and the time spent while holding the wire (86%) in the rotarod and grip strength tests, respectively, as compared to control group; *F*(3, 56) = 266.8 and 284.3, respectively (*p* < 0.0001). Diapocynin administration alleviated these motor abnormalities as manifested by the marked increase in the total distance traveled (4.6-fold), mean speed (5.9-fold), rearing frequency (17-fold), distance and time active in the center (3- and 3.9-fold, respectively) as well as the periphery (3.5- and 1.5-fold, respectively), rotarod fall off latency (10.2-fold) and time spent holding the wire (4.2-fold) together with diminished immobility time (28%), as related to 3-NP group (*p* < 0.0001). Normal rats which received diapocynin only did not provoke any significant alterations in locomotor activity, motor co-ordination, or muscle strength, as compared with normal control animals (Figs. 1, 2).

### Diapocynin attenuated oxidative stress induced by 3-NP injection in rats

**Fig. 3:**
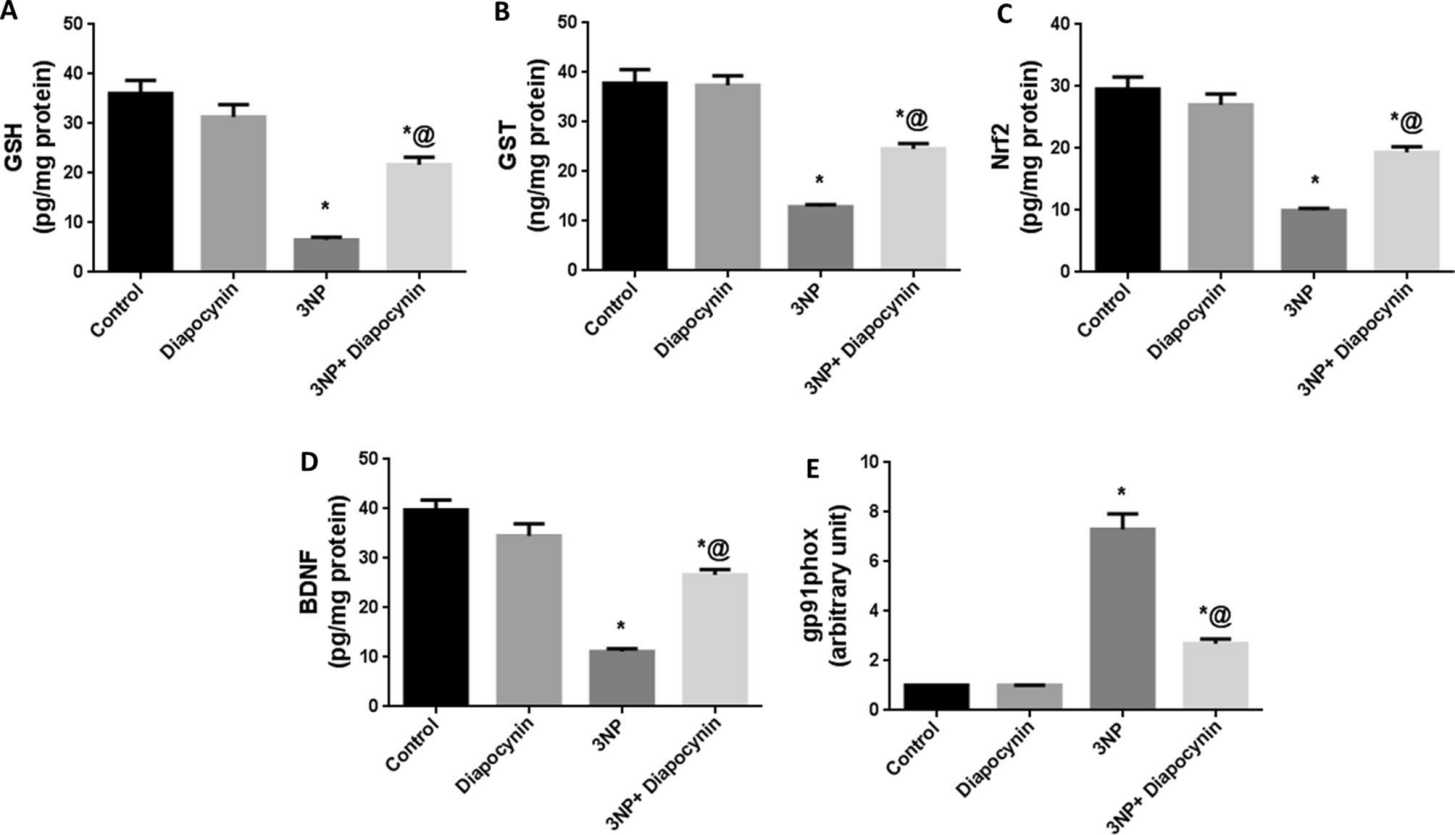
Effect of diapocynin on 3-NP-induced oxidative stress. **A** GSH, **B** GST, **C** Nrf2, **D** BDNF striatal contents, and **E** gp91phox gene expression. Data were expressed as mean ± S.E.M. (*n* = 6). Statistical analysis was done using one-way ANOVA followed by Tukey’s post hoc test. ^*^p < 0.05 vs. control, ^@^*p* < 0.05 vs. 3NP group. *3-NP* 3-nitropropionic acid, *GSH* reduced glutathione, *GST* glutathione-S-transferase, *Nrf2* nuclear factor erythroid 2-related factor 2, *BDNF* brain-derived neurotrophic factor

Rats which received 3-NP injection displayed augmented oxidative stress with significant increase in gene expression of gp91phox; the major subunit of NOX2 by 7.2-fold, while GSH, GST, Nrf2, and BDNF striatal contents were markedly reduced by 82, 66, 66, and 72%, respectively, in comparison with control group rats. Diapocynin attenuated 3-NP-induced oxidative hazards with obvious elevation in the defensive mechanisms namely; GSH (3.3-fold), GST (1.9-fold), Nrf2 (1.9-fold), and BDNF (2.4-fold), whereas gp91phox expression was markedly reduced by 63% with regards to 3-NP group, F(3, 20) = 43.73, 45.41, 39.29, 54.36, and 82.39, respectively (*p* < 0.0001). Administration of diapocynin to normal animals did not induce any significant changes in oxidative stress markers, as compared to normal control group (Fig. 3).

### Diapocynin inhibited neuro-inflammation induced by 3-NP injection in rats

**Fig. 4:**
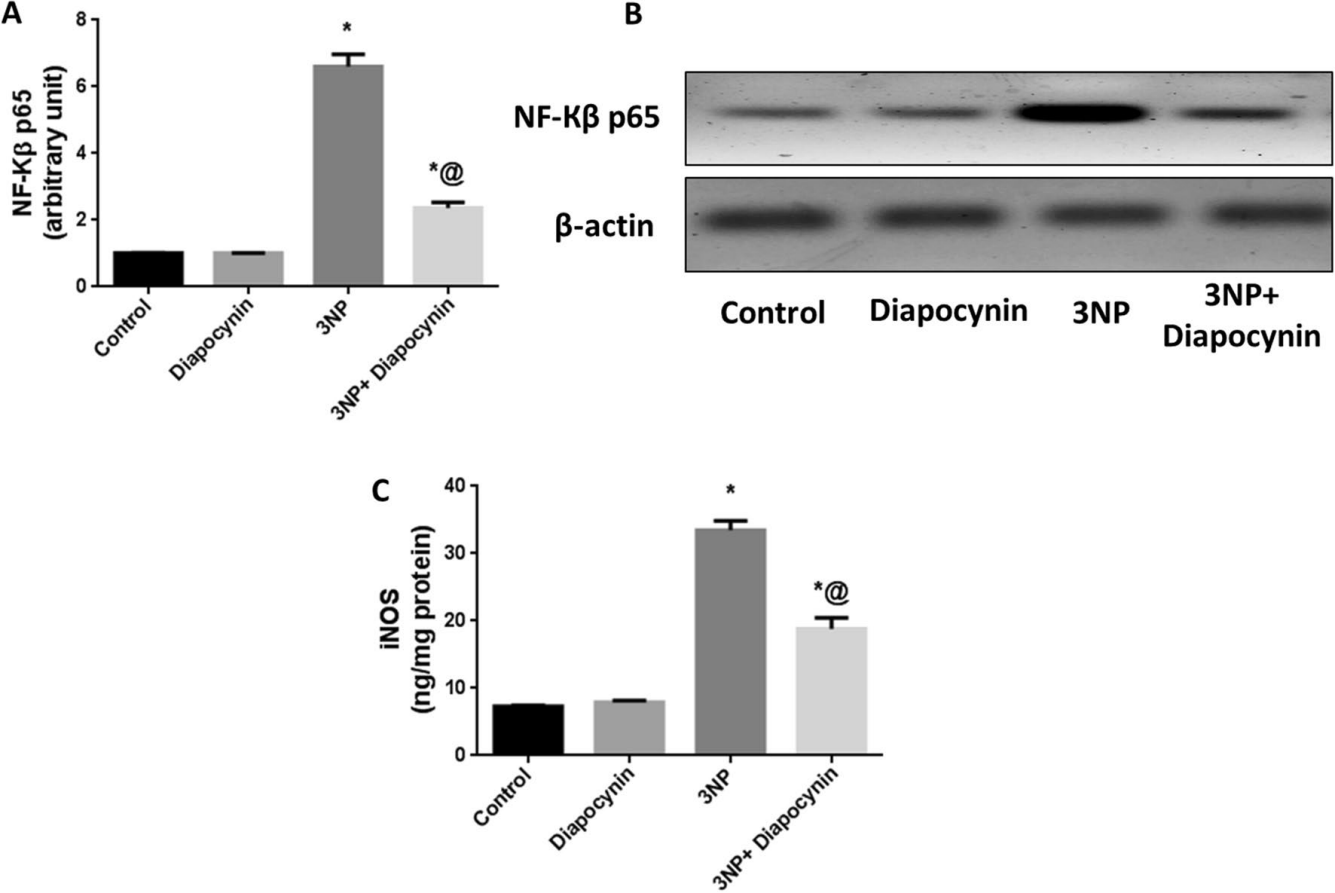
Effect of diapocynin on 3-NP-induced neuro-inflammation. **A** NF-Кβ p65 expression, **B** Western blot of NF-Кβ p65, and **C** iNOS striatal content. Data were expressed as mean ± S.E.M. (*n* = 6). Statistical analysis was done using one-way ANOVA followed by Tukey’s post hoc test. ^*^p < 0.05 vs. control, ^@^*p* < 0.05 vs. 3NP group. *3-NP* 3-nitropropionic acid, *NF-Кβ* nuclear factor-Кβ, *iNOS* inducible nitric oxide synthase

3-NP injection induced marked increase in iNOS striatal content along with significant upregulation of NF-Кβ p65 by 4.6- and 6.5-fold, respectively, as compared with control group. Treatment with diapocynin ensued a noticeable inhibition in NF-Кβ p65 expression (64%) as well as marked reduction in iNOS content (44%) in comparison with 3-NP group, *F*(3, 20) = 167.1 and 127.4, respectively (*p* < 0.0001). Normal rats which received diapocynin showed no significant changes in the formerly mentioned parameters, as compared to the normal control animals (Fig. 4).

### Diapocynin mitigated the exaggerated apoptosis induced by 3-NP injection in rats

**Fig. 5:**
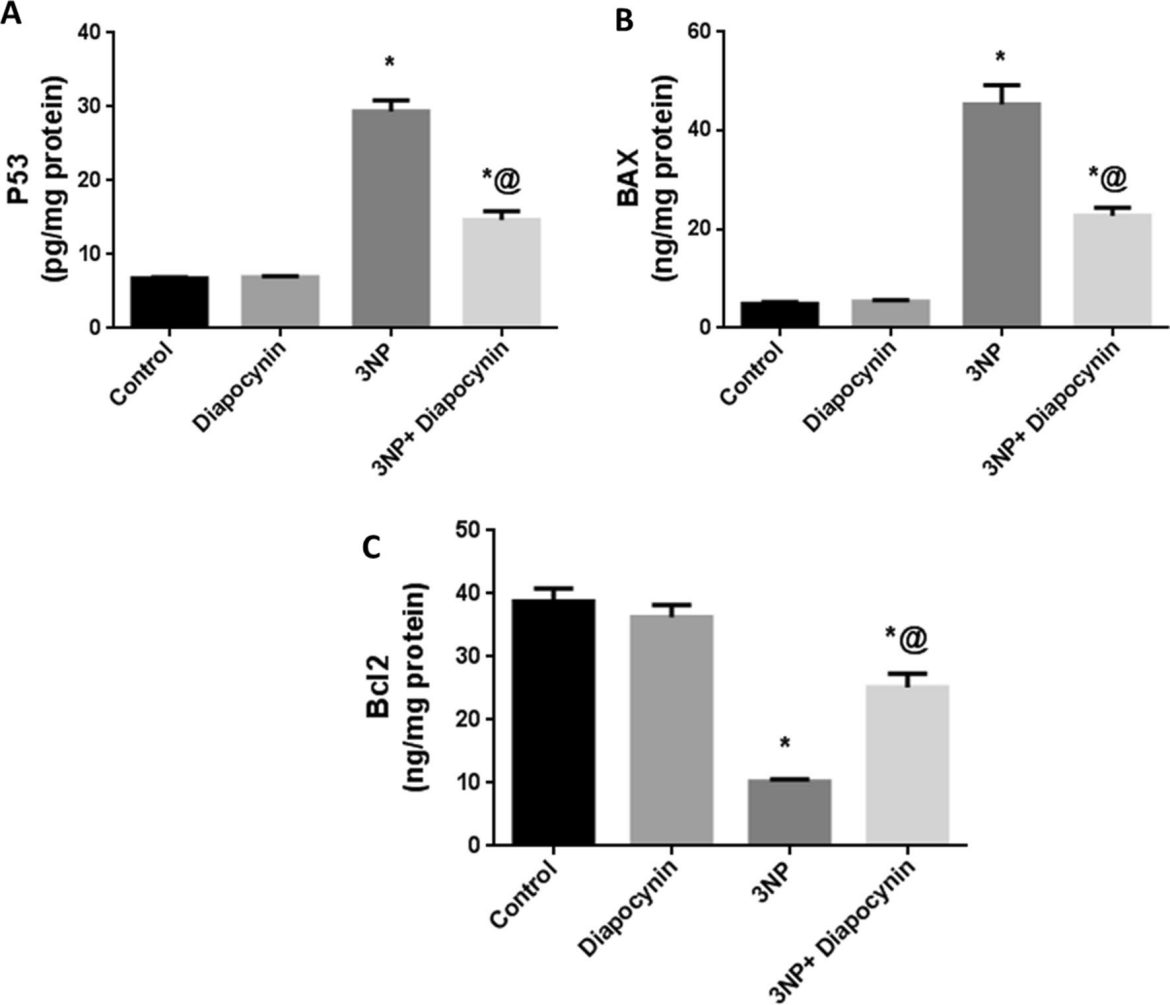
Effect of diapocynin on 3-NP-associated apoptosis. **A** P53, **B** BAX, and **C** Bcl2 striatal contents. Data were expressed as mean ± S.E.M. (*n* = 6). Statistical analysis was done using one-way ANOVA followed by Tukey’s post hoc test. ^*^p < 0.05 vs. control, ^@^*p* < 0.05 vs. 3NP group. *3-NP* 3-nitropropionic acid, *P53* tumor suppressor protein, *BAX* Bcl-2-associated X protein, *Bcl2* B-cell lymphoma-2

The striatal contents of P53 as well as BAX which are key players in the apoptotic pathway were markedly increased in 3-NP-injected rats by 4.3- and 9.2-fold, respectively, with regards to normal control animals. However, the anti-apoptotic; Bcl2 content was significantly reduced upon 3-NP injection by 74%, in comparison with control group. Diapocynin mitigated 3-NP-induced apoptosis with noticeable decrease in P53 (50%) and BAX (50%) contents on the contrary to Bcl2 increased striatal content by 2.5-fold, *F*(3, 20) = 121.6, 80.09, and 51.81, respectively (*p* < 0.0001). Diapocynin administration in normal animals did not induce significant changes in the previously mentioned apoptotic parameters, in comparison with the control group rats (Fig. 5).

### Diapocynin diminished 3-NP-induced alterations in Sirt1 protein expression

**Fig. 6:**
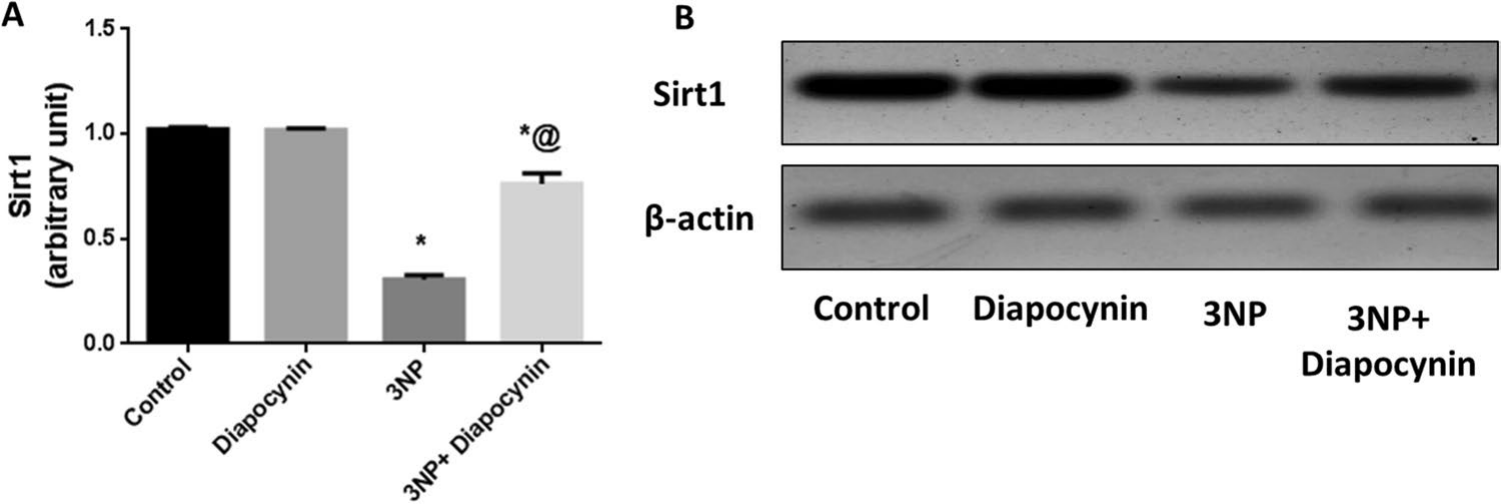
Effect of diapocynin administration on 3-NP-induced alterations in **A** Sirt1 expression and **B** Western blot of Sirt1. Data were expressed as mean ± S.E.M. (*n* = 6). Statistical analysis was done using one-way ANOVA followed by Tukey’s post hoc test. ^*^p < 0.05 vs. control, ^@^*p* < 0.05 vs. 3NP group. *3-NP* 3-nitropropionic acid, *Sirt1* silent information regulator 1

3-NP injection was accompanied with prominent downregulation of Sirt1 protein expression by 70% as compared with the control group. Treatment with diapocynin mitigated 3-NP-provoked Sirt1 depression leading to significant increase in its protein level by 2.5-fold, *F*(3, 20) = 141.1 (*p* < 0.0001). Normal animals which received diapocynin revealed no significant changes in the expression of Sirt1 in comparison with the normal control group (Fig. 6).

### Diapocynin attenuated 3-NP-induced histopathological alterations in striatum

**Fig. 7:**
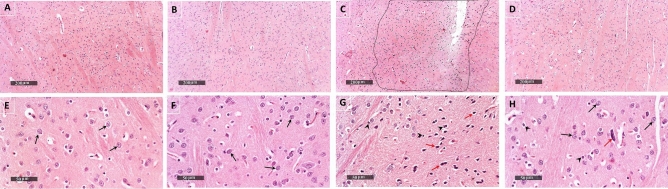
Effect of diapocynin administration on 3-NP-induced histopathological alterations. Representative H&E photomicrographs (*n* = 3) of control group (**A**, **E**), diapocynin group (**B**, **F**), 3-NP group (**C**, **G**), and 3-NP + diapocynin group (**D**, **H**). *Black arrows* indicate intact neurons whereas *red arrows* represent degenerated ones. Glial cells infiltration was demonstrated by *arrow heads*. Magnifications: ×100 (**A–D**) and ×400 (**E–H**). *3-NP* 3-nitropropionic acid

**Fig. 8:**
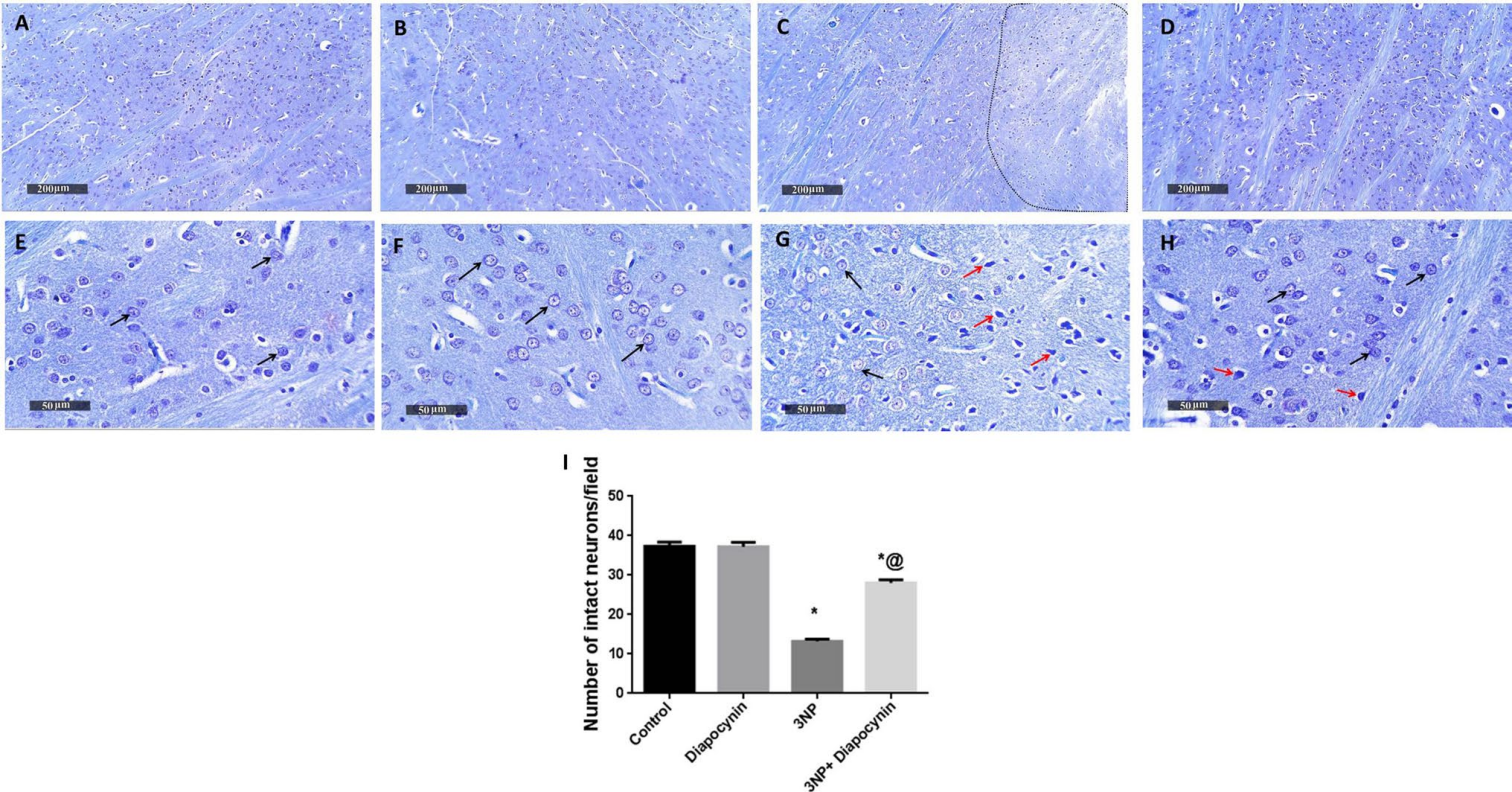
Effect of diapocynin administration on neuronal survival in 3-NP-injected rats. Illustrative Nissl-stained photomicrographs of control group (**A**, **E**), diapocynin group (**B**, **F**), 3-NP group (**C**, **G**), and 3-NP + diapocynin group (**D**, **H**). *Black arrows* indicate intact neurons, whereas *red arrows* represent degenerated ones. Magnifications: ×100 (**A–D**) and ×400 (**E–H**). **I** A bar chart representing the mean number of intact neurons in each group with each bar with vertical line illustrating the mean ± S.E.M. of 3 rats per group. Data were analyzed using one-way ANOVA followed by Tukey’s post hoc test. ^*^ *p* < 0.05 vs. control, ^@^*p* < 0.05 vs. 3NP group. *3-NP* 3-nitropropionic acid

Striatal tissues were examined by H&E in addition to Nissl stain which was used to determine the number of intact neurons. Photomicrographs of normal animals demonstrated normal morphological features of striatal regions with many records of apparent intact neurons of different sizes with intact subcellular and nuclear details (black arrows), in addition to showing intact intercellular brain matrix with minimal records of reactive glial cells infiltrates. Diapocynin administration in normal animals revealed almost the same records as the control group without abnormal histological alterations. 3-NP-injected rats demonstrated a focal area at anterior lateral border of striatal regions showing many records of darkly stained and pyknotic degenerated neurons (red arrows). Photomicrographs of 3-NP group also revealed significant neuronal loss and moderate edema of brain matrix accompanied with many reactive microglial cells infiltrates in lesion core and higher reactive astrocytic infiltrates allover striatal regions and around core lesion borders (arrow heads). Treatment with diapocynin induced significant neuroprotection with abundant records of apparent intact neurons (black arrows) and few scattered degenerated cells (red arrows) along with intact intercellular brain matrix with significantly fewer reactive glial cells infiltrates (arrow heads) (Figs. 7 and 8).

### Diapocynin suppressed 3-NP-induced immunohistochemical expression of Iba1 and GFAP

**Fig. 9:**
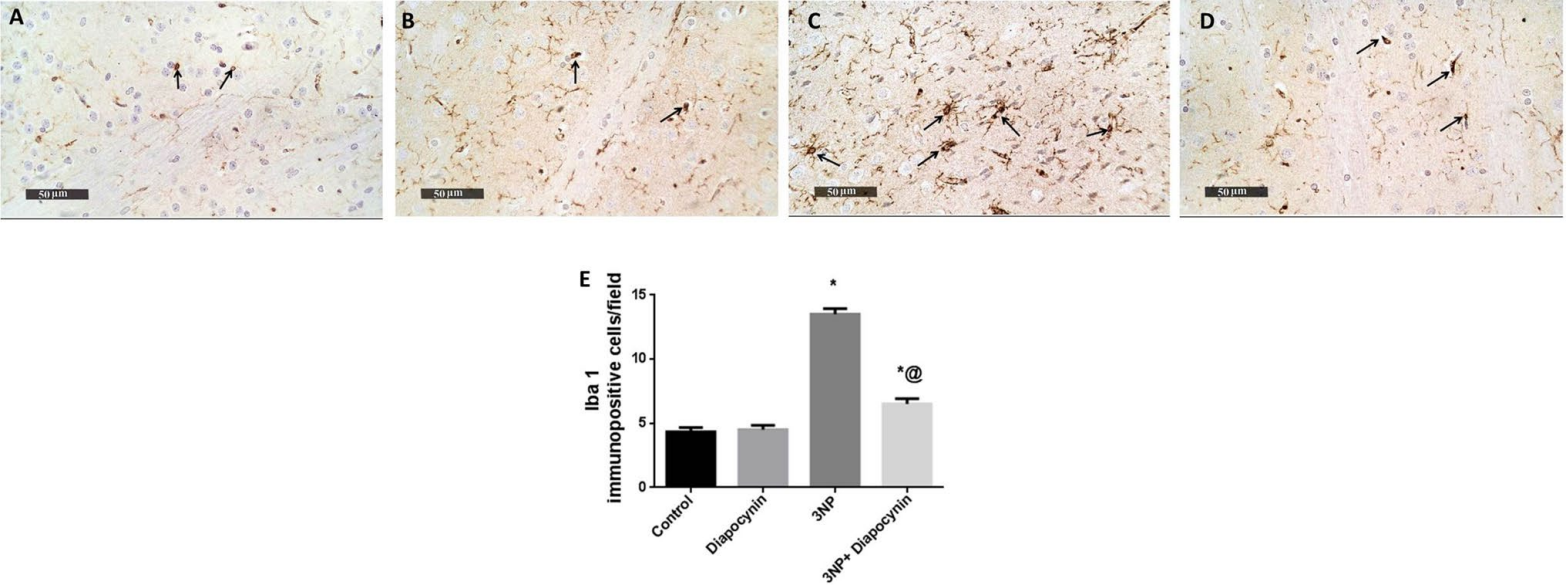
Effect of diapocynin administration on 3-NP-induced immuno- histochemical expression of Iba1. Photomicrographs of control group (**A**), diapocynin group (**B**), 3-NP group (**C**), and 3-NP + diapocynin group (**D**). *Black arrows* indicate Iba1 immunoexpression. Magnification: ×400 (**A–D**). **E** A bar chart representing the mean number of Iba1-positive cells in each group with each bar with vertical line illustrating the mean ± S.E.M. of 3 rats per group. Data were analyzed using one-way ANOVA followed by Tukey’s post hoc test. ^*^p < 0.05 vs. control, ^@^*p* < 0.05 vs. 3NP group. *3-NP* 3-nitropropionic acid, *Iba1* ionized calcium binding adaptor molecule 1

**Fig. 10:**
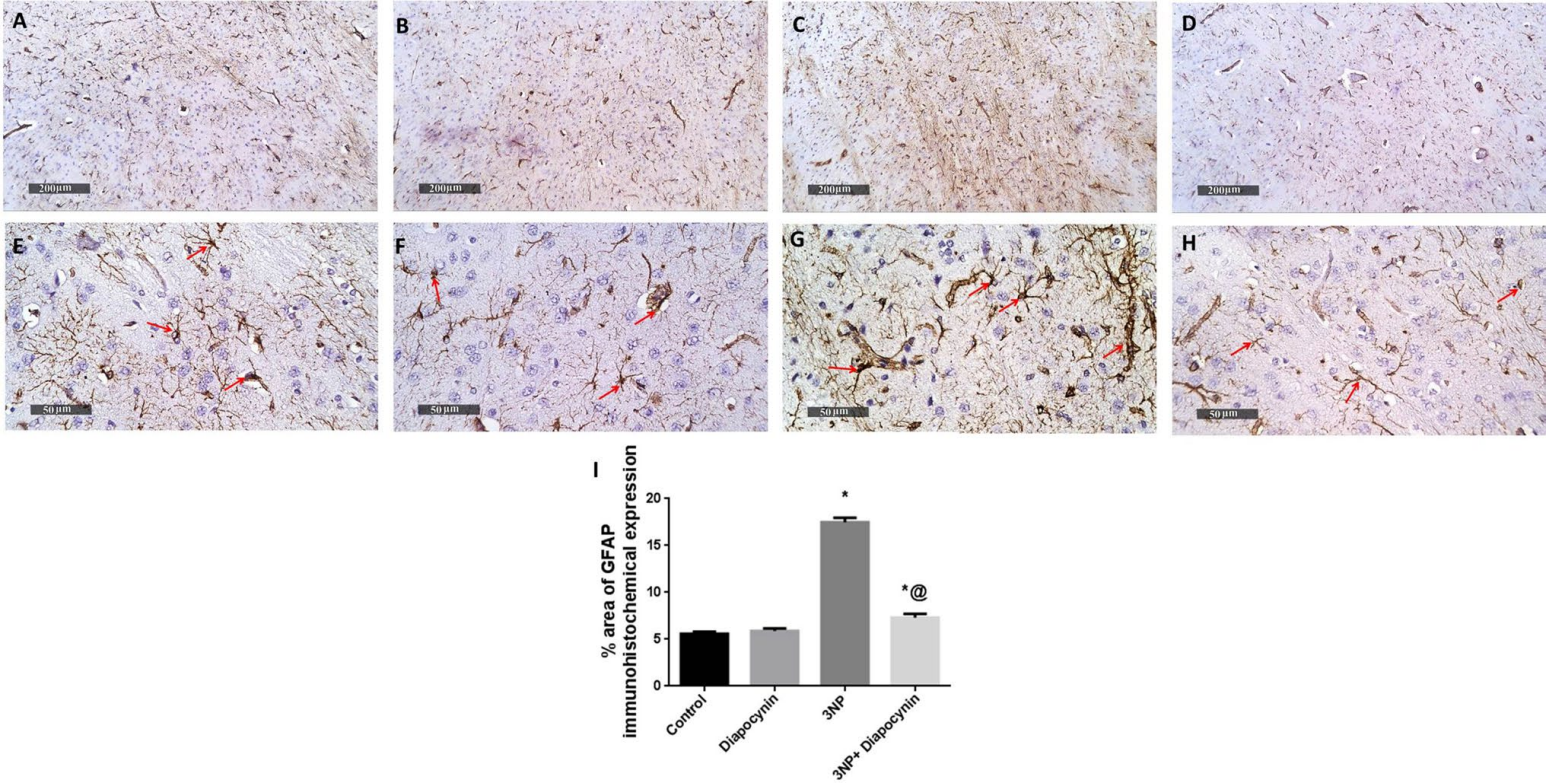
Effect of diapocynin administration on 3-NP-induced immunohistochemical expression of GFAP. Photomicrographs of control group (**A**, **E**), diapocynin group (**B**, **F**), 3-NP group (**C**, **G**), and 3-NP + diapocynin group (**D**, **H**). *Black arrows* indicate GFAP immunoexpression. Magnifications: ×100 (**A–D**) and ×400 (**E–H**). **I** A bar chart representing the mean percentage of GFAP immunoexpression in each group with each bar with vertical line illustrating the mean ± S.E.M. of 3 rats per group. Data were analyzed using one-way ANOVA followed by Tukey’s post hoc test. ^*^p < 0.05 vs. control, ^@^*p* < 0.05 vs. 3NP group. *3-NP* 3-nitropropionic acid, *GFAP* glial fibrillary acidic protein

3-NP provoked microglial and astroglial activation with subsequent rise in Iba1-positive microglial cells (3.1-fold) and % area of GFAP immunoexpression (3.1-fold), respectively. Diapocynin administration seized 3-NP-associated gliosis with prominent reduction in Iba1 and GFAP immunoreactivity by 52 and 58%, respectively, *F*(3, 20) = 124.9 and 245 (*p* < 0.0001). In normal rats, diapocynin did not induce any abnormal changes regarding both markers immunohistochemical expression, as compared to normal control group (Figs. 9 and 10).

## Discussion

The current investigation emphasized the neuroprotective effects rendered by diapocynin, a selective NOX inhibitor, against 3-NP neurotoxicity model of HD. It was previously stated that 3-NP injection provokes striatal neurodegeneration resulting in prominent dysfunction in animals' grip strength and locomotor activities as well as motor co-ordination (Sayed et al. 2020). Correspondingly, in the present study, 3-NP-injected rats displayed diminished grip strength, impaired motor co-ordination, and disrupted locomotor function in grip strength, rotarod, and open field tests, respectively. Remarkably, diapocynin administration improved 3-NP-injected rats' grip strength and locomotor behavior in the previously mentioned corresponding tests as well as their ability to stay for a longer time during the rotarod assessment. In context, diapocynin was previously reported to significantly restore locomotor and motor co-ordination impairments in MPTP-induced Parkinson's disease mouse model (Ghosh et al. 2012). It also rescued motor dysfunction induced in organophosphate-intoxicated rats (Putra et al. 2020).

In the current study, 3-NP-injected animals exhibited a state of oxidative stress as indicated by prominent reduction in GSH and GST contents contrary to increased gp91phox; NOX2 subunit expression. In fact, the mitochondrial toxin 3-NP is reported to penetrate the blood brain barrier and produce surplus of ROS leading to pathological symptoms simulating that associated with HD (Gonchar et al. 2021). The increased oxidative state and neuronal degeneration triggered by 3-NP injection might be attributed, in part, to the obvious suppression of BDNF/Nrf2 anti-oxidant machinery as reported herein. Nrf2 is known to modulate the production of GSH and its related enzymes as GST; thus, it could overcome the augmented oxidative stress associated with 3-NP (Vasconcelos et al. 2019). Moreover, Nrf2 inhibits NOX activity, so it hinders ROS production via this fundamental enzyme in ROS generation (Benarroch 2017). Nrf2 is also reported to induce transcription of BDNF, an important neurotrophic factor which is involved in neuronal proliferation, differentiation, and survival as well as memory and learning functions (Huang et al. 2020; Yao et al. 2021). Meanwhile, BDNF is implicated in Nrf2 translocation and consequent activation leading to repaired redox hemostasis (Bouvier et al. 2017). It was previously reported that BDNF protein levels are reduced in HD animal models (Ferrer et al. 2000; Duan et al. 2003). A prominent decrease of GSH as a result of Nrf2 downregulation was also previously detected in 3-NP model in rats (Sayed et al. 2020). Concurrently, HD is associated with augmented NOX2 activity leading to massive ROS generation and cell death (Valencia et al. 2013). On the other hand, administration of diapocynin in the present study alleviated 3-NP-associated oxidative hazards with significant enhancement in the previously mentioned anti-oxidant defenses, namely BDNF, Nrf2, GSH, and GST, whereas gp91phox expression was noticeably reduced.

Nrf2 signaling pathway is also reported to inhibit neuro-inflammation induced by the redox sensitive transcription factor; NF-Кβ which contributes to neuronal injury linked to 3-NP injection (Brandes and Gray 2020). NF-Кβ is also known to induce microglial activation with consequent release of pro-inflammatory mediators as iNOS which promotes cell death via nitric oxide deleterious hazards (Napolitano et al. 2008). In accordance with these findings, 3-NP-injected animals, in the present investigation, have shown prominent upregulation of NF-Кβ p65 along with increased iNOS content while upon treatment with diapocynin, redox status was improved with reduced iNOS striatal content owing to NF-Кβ p65 downregulation.

Sirt1, a nicotinamide adenine dinucleotide-dependent enzyme, is a critical key player in the regulation of oxidative stress, inflammation, and apoptosis (Ren et al. 2019). It is reported that Sirt1 can inhibit NF-Кβ p65 activation thus, combatting its associated inflammatory milieu (Yeung et al. 2004). Additionally, Sirt1 is involved in apoptosis regulation via inhibition of P53 with consequent downregulation of the apoptotic protein; BAX (Vaziri et al. 2001). Moreover, Sirt1 induces the expression of Nrf2 anti-oxidative pathway, consequently inhibition of Sirt1 is implicated in neuro-inflammation, apoptosis as well as oxidative stress (Huang et al. 2015). Interestingly, Sirt1 can also modulate neuro-inflammation via reducing astrocyte activation with subsequent decrease in GFAP; a well-known marker of astrogliosis (Shaheen et al. 2021). Additionally, Sirt1 was reported to inhibit microglial activation and its deleterious inflammatory cascade (Li et al. 2020). In the current study, rats receiving 3-NP have shown increased NF-Кβ p65 expression as well as P53 up-leveling with consequent elevation in BAX content while Bcl2 content was obviously reduced. These findings could be attributed to down regulation of Sirt1 and Nrf2 inhibition detected in that group. Diapocynin enhanced Sirt1/Nrf2 expression with consequent downregulation of NF-Кβ p65 in addition to decreased P53 level associated with marked elevation in the anti-apoptotic Bcl2 content contrary to decreased pro-apoptotic BAX striatal content. Besides, Sirt1 downregulation was accompanied by prominent increments of Iba1-positive microglial cells as well as GFAP immunoreactivity which indicate microglial and astrocytic activation witnessed in 3-NP group, respectively. However, diapocynin administration attenuated glial reactivity as a reflection of dismounted gliosis and inflammation through Sirt1 induction. Thus, diapocynin diminished 3-NP-associated oxidative stress, inflammation, gliosis, and apoptosis via augmenting Sirt1/Nrf2 pathway.

Finally, 3-NP is implicated in apparent pyknosis with marked decrease in surviving neurons evidenced by Nissl stain (Gao et al. 2015). Animals receiving 3-NP, in the present investigation, have shown noticeable neuronal pyknosis with marked increase in darkly stained degenerated neurons which indicates an evident decline in neuronal survival. Interestingly, 3-NP-instigated histopathological anomalies were mitigated upon diapocynin administration as indicated by noticeable increase in the number of intact neurons.

## Conclusion

The present study highlights the neuroprotection rendered by diapocynin in 3-NP-instigated HD model in rats proposing Sirt1/Nrf2 pathway modulation as a key player in its beneficial action against oxidative stress, neuro-inflammation, and apoptosis. Consequently, diapocynin is a profound nominee for HD management.

## Data Availability

The original contributions presented in the study are included in the article, further inquiries can be directed to the corresponding author.

## Abbreviations

HD: Huntington's disease
3-NP: 3-Nitropropionic acid
Sirt1: Silent information regulator 1
NOX: Nicotinamide dinucleotide phosphate (NADPH) oxidase
ROS: Reactive oxygen species
Nrf2: Nuclear factor erythroid 2-related factor 2
NF-Кβ: Nuclear factor-Кβ
GSH: Reduced glutathione
BDNF: Brain-derived neurotrophic factor
P53: Tumor suppressor protein
BAX: Bcl-2-associated X protein
MPTP: 1-Methyl-4-phenyl-1,2,3,6-tetrahydropyridine
GST: Glutathione-S-transferase
iNOS: Inducible nitric oxide synthase
Bcl2: B-cell lymphoma-2
Iba1: Ionized calcium binding adaptor molecule 1
GFAP: Glial fibrillary acidic protein
